# Ru(II)(*ƞ*^6^-*p*-cymene) Conjugates Loaded onto Graphene Oxide: An Effective pH-Responsive Anticancer Drug Delivery System

**DOI:** 10.3390/molecules27217592

**Published:** 2022-11-05

**Authors:** Suffora Akhter, Farukh Arjmand, Claudio Pettinari, Sartaj Tabassum

**Affiliations:** 1 Department of Chemistry, Aligarh Muslim University, Aligarh 202002, India; 2 School of Pharmacy, University of Camerino, Via S. Agostino 1, 62032 Camerino, MC, Italy

**Keywords:** Ru(II)(*ƞ*6-*p*-cymene) conjugates, graphene oxide loading, ultrasonication, binding studies with ct-DNA, cytotoxicity

## Abstract

Graphene oxide-based nanodrug delivery systems are considered one of the most promising platforms to deliver therapeutic drugs at the target site. In this study, Ru(II)(*ƞ*6-*p*-cymene) complexes containing the benzothiazole ligand were covalently anchored on graphene oxide using the ultrasonication method. The nanoconjugates GO-NCD-**1** and GO-NCD-**2** were characterized by FT-IR, UV-visible, ^1^**H** NMR, TGA, SEM, and TEM techniques, which confirmed the successful loading of both the complexes (NCD **1** and NCD **2**) on the carrier with average particle diameter sizes of 17 ± 6.9 nm and 25 ± 6.5 nm. In vitro DNA binding studies of the nanoconjugates were carried out by employing various biophysical methods to investigate the binding interaction with the therapeutic target biomolecule and to quantify the intrinsic binding constant values useful to understand their binding affinity. Our results suggest (i) high *K*_b_ and *K*_sv_ values of the graphene-loaded conjugates (ii) effective cleavage of plasmid DNA at a lower concentration of 7.5 µM and 10 µM via an oxidative pathway, and (iii) fast release of NCD **2** at an acidic pH that could have a good impact on the controlled delivery of drug. It was found that 90% of the drug was released in an acidic pH (5.8 pH) environment in 48 h, therefore suggesting pH-responsive behavior of the drug delivery system. Molecular docking, DFT studies, and cytotoxicity activity against three cancer cell lines by SRB assay were also performed.

## 1. Introduction

Cancer is defined as the uncontrolled growth and replication of cells, which eventually develop into lumps or masses known as tumors. These tumor cells could migrate from the primary site of origin to distant tissues via blood or lymph to cause secondary cancers, termed ‘metastasis’ [1]. Cancer is the most lethal disease globally, with a death toll of 10 million in 2020, reported by the WHO, which is expected to rise exponentially to 18 million in 2030 [2]. Chemotherapy is widely used for treating metastatic cancer, either alone or in combination with surgery or radiotherapy. Since, there are many phenotypes of cancer arising from multiple genetic or epigenetic alterations of cancer types, most of the chemotherapeutic drugs do not act broadly across different tumor types [3,4,5]. Classical metal-based chemotherapeutic agents manifest adverse effects such as dose-limiting side effects, reduced bioavailability of the drugs at the target site, and a low therapeutic response, which could be due to various factors such as lower aqueous solubility, less stability, fast metabolism, and non-selective drug distribution [6,7,8]. Innovative strategies have led to the evolution of drug delivery vehicles, which are smart therapeutic systems that react ‘intelligently’, and can overcome the biological barriers to selectively distinguish healthy cells from cancerous ones, and can release therapeutic agents on-demand at the optimal dosage range [9]. Different nanomaterials have been used as nanocarriers in healthcare to modulate the pharmacokinetic profile of chemotherapeutic agents and to reduce the side effects of chemotherapy by selective and targeted delivery of these anti-cancer therapeutics [10,11]. These nanocarriers have been developed from various inorganic and organic components fabricated in different architectures, *viz.,* polymeric micelles, nanoparticles, liposomes, and dendrimers, which can carry the active cargo easily to the target [12].

Graphene oxide (GO) has been studied intensively for clinical drug delivery practice due to several advantages, such as relevant specific surface area, exceptional physical and chemical properties, better biosafety, and ease of modification [13,14]. GO is a sheet of graphene modified with various functional groups, like carboxyl (−COOH), hydroxyl (−OH), and epoxy (−O−) which are responsible for its hydrophilicity and therefore could improve the solubility of some water-insoluble drugs and make it an efficient drug delivery system [15,16]. Furthermore, GO can be used as a controlled release platform that releases drugs in a predictable, pre-set persistent manner to maintain a constant drug concentration for an extended period with a single dosage, which ultimately would result in fewer adverse effects [17]. Liu et al. reported that the water solubility of an insoluble drug, SN38, was improved upon its encapsulation in GO functionalized with polyethylene glycol (PEG), which in turn increased its anticancer response [18]. Similarly, the loading of platinum-based anticancer drugs such as cisplatin, carboplatin, and oxaliplatin, etc. on polyethylene glycol, folic acid, chitosan, and high polymer polyethyleneimine grafted onto graphene oxide were demonstrated to achieve effective delivery of these drugs with lower toxicity.

Literature reports have revealed that ruthenium-based chemotherapeutic drugs exhibit interesting advantages over cisplatin, including reduced toxicity in cancer cells as demonstrated by their binding profiles to DNA, enzymes, and protein active sites [19,20]. Nevertheless, the poor aqueous solubility of ruthenium drug candidates such as *fac*-[Ru(Cl)_3_(NH_3_)_3_] prevented their clinical use [21]. In the present study, we have loaded two previously synthesized Ru(II)(*ƞ*6-*p*-cymene)-based drug candidates, NCD **1** and NCD **2,** onto the nanocarrier graphene oxide by ultrasonication. The nanoconjugate drugs NCD **1** and NCD **2** contain the ligands 2-amino-4-cholorobenzothiazole and 2-amino-6-fluorobenzothiazole, which are coordinated to the metal centre in a typical ‘three-legged piano stool’ geometry. The successful loading and binding of these nanoconjugates were validated by different spectroscopic techniques. Various biophysical techniques were employed to investigate the influence of the substituted halogens on the DNA binding interaction and anticancer activity of these drug conjugates after their successful encapsulation on the nanocarrier [22]. The controlled release behavior of these potential drugs was studied at different pH levels. Further, the cytotoxicity activity was evaluated against three cancer cell lines by SRB assay to validate their potency by using an effective drug delivery system, graphene oxide.

## 2. Results and Discussion

### 2.1. Synthesis and Characterization

**Scheme 1:**
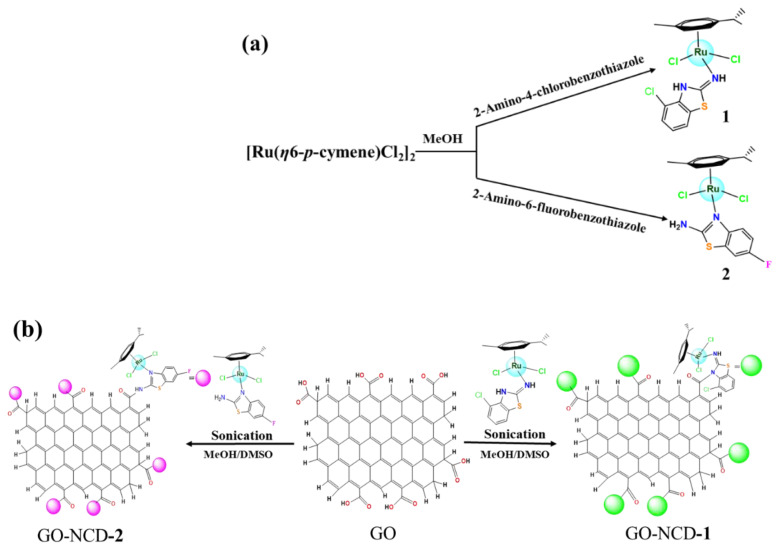
Synthetic route showing the synthesis of (**a**) NCD **1** and NCD **2** (**b**) GO-NCD-**1** and GO-NCD-**2**.

Two ruthenium-based drug conjugates were synthesized by methods previously reported by our group [23]. Both of the complexes were encapsulated on graphene oxide by ultrasonication. A schematic representation of the synthesis of the ruthenium-based nanoconjugates and graphene oxide loaded ruthenium conjugates GO-NCD-**1** and GO-NCD-**2** is shown in Scheme 1a,b, respectively [24].

**Figure 1:**
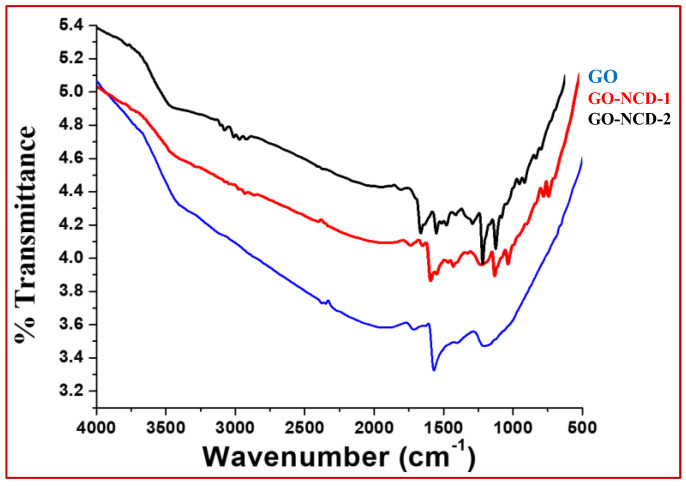
IR spectra of GO, GO-NCD-**1,** and GO-NCD-**2**.

GO-NCD-**1** and GO-NCD-**2** were characterized by employing IR spectroscopy, as depicted in Figure 1. The infrared spectrum of GO-NCD-**1** displayed a band at 3221.00 cm^−1^ labelled to the υ–(NH) stretching frequency. The infrared spectrum of GO-NCD-**2** displayed a band at 3225.47 cm^−1^ suggesting the presence of –NH. Υ–(CH_2)_ and υ(–C=N) vibrations were found at 2966.96 cm^−1^ and 1610.49 cm^−1^ in the free complexes and were also observed in the loaded composites. The IR spectrum of GO exhibited bands at 3439 cm^−1^, 1720 cm^−1^, 1568.19 cm^−1^, and 1199.6 cm^−1^ corresponding to the –OH stretching vibrations, –C=O stretching vibrations, C=C stretching vibrations, and C–O stretching vibrations, respectively. Those additional signals in the composites indicate the successful anchoring of both complexes onto the graphene sheet.

**^1^H** NMR spectra of the NCD **1**, NCD **2**, GO-NCD-**1,** and GO-NCD-**2** were obtained to get more information about the conjugation of drugs on the carrier and are given in Figures S10 and S11, Supplementary Materials. The characteristic peaks of NCD **1** and NCD **2** showed a slight shift after their loading on to the nanocarrier, which suggested successful encapsulation of these drugs on the nanocarrier. The peak around 10 ppm corresponding to the carboxyl protons of the carrier, is absent in the loaded composites, therefore confirming the successful loading of the drug conjugates. Extra peaks in the region of 2–3 ppm were observed in the loaded composites, which could be assigned to the protons of the graphene oxide probably shielded by the diamagnetic ring currents [25,26].

**Figure 2:**
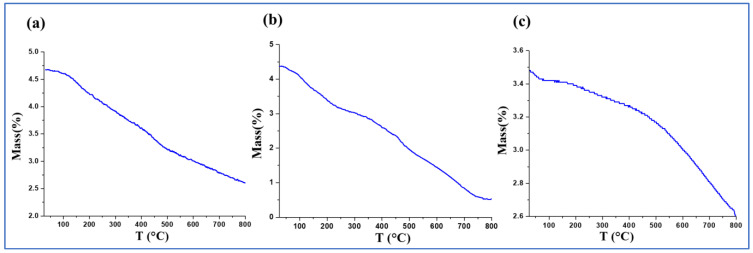
TGA of (**a**) GO-NCD-**1** (**b**) GO-NCD-**2,** and (**c**) GO.

Thermal gravity analysis of the GO, GO-NCD-**1,** and GO-NCD-**2** composites was carried out to understand their decomposition behavior. The important mass loss events that occur in TGA are <100 °C for the elimination of water, 100–350 °C for the loss of oxygen containing functional groups, and 350–1000 °C for the oxidation pyrolysis of carbon framework [27]. GO-NCD-**1** and GO-NCD-**2** showed loss of mass at 150, 250, 350, and 500 °C therefore confirming the successful loading of both the complexes on graphene oxide. The plot in Figure 2 shows, in fact, that GO is thermally stable up to 450 °C, whereas both nanocomposites containing the Ru species are found to be thermally less stable, likely due to the loss of CO_2_ and N_2_ (Figure 2, Figures S7 and S8).

**Figure 3:**
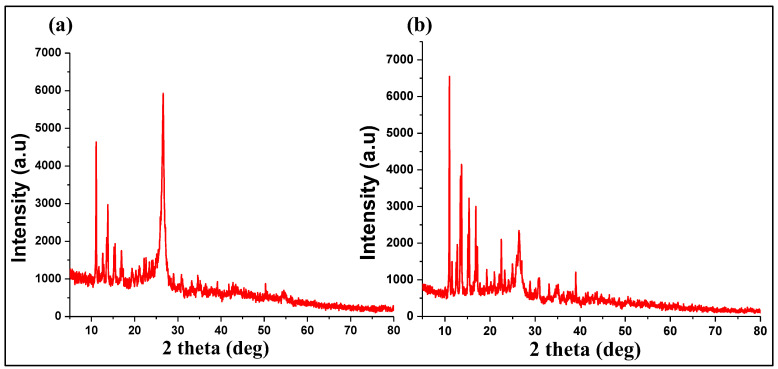
Simulated powder X-ray diffraction patterns of (**a**) GO-NCD-**1** and (**b**) GO-NCD-**2**.

The XRD spectra were measured in the range of 5 to 80°. In the case of GO, peaks were observed at 2*θ* = 11.11°, 26.59°, 13.9°, and 10.54°, whereas GO-NCD-**1** showed peaks at 13.82°, 15.46°, 16.81°, and 26.4°. For GO-NCD-2, peaks were observed at 10.84°, 13.56°, 15.04°, 16.04°, 22.37°, 26.59°, and 38.95°. The peaks at 11.11° were assigned to the oxygenic functional groups and trapped water molecules of the graphene oxide. The broadening of the peaks of NCD **1** and NCD **2** were observed after their loading on graphene oxide, which could be due to the decrease in size from bulk to nanoscale dimensions (Figure 3, Figures S4 and S5). The broadening of the peaks around 26.59° suggested the functionalization of the major oxygen containing groups of the GO, therefore confirming the successful binding of both drug candidates on the nanodrug carrier [28,29,30].

**Figure 4:**
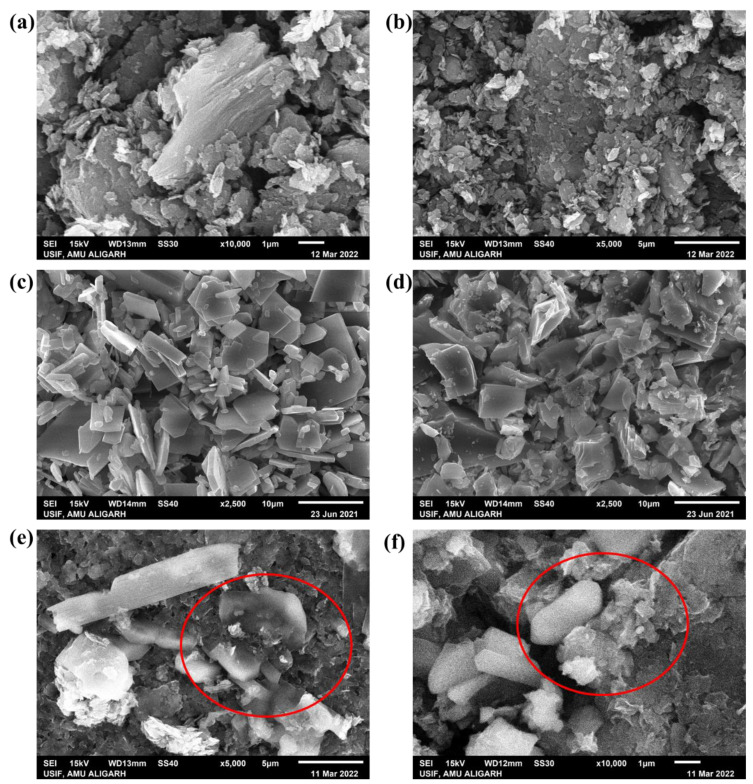
SEM images of GO (**a**,**b**) NCD-**1** (**c**) NCD-**2** (**d**) GO-NCD-**1** (**e**) GO-NCD-**2** (**f**).

**Figure 5:**
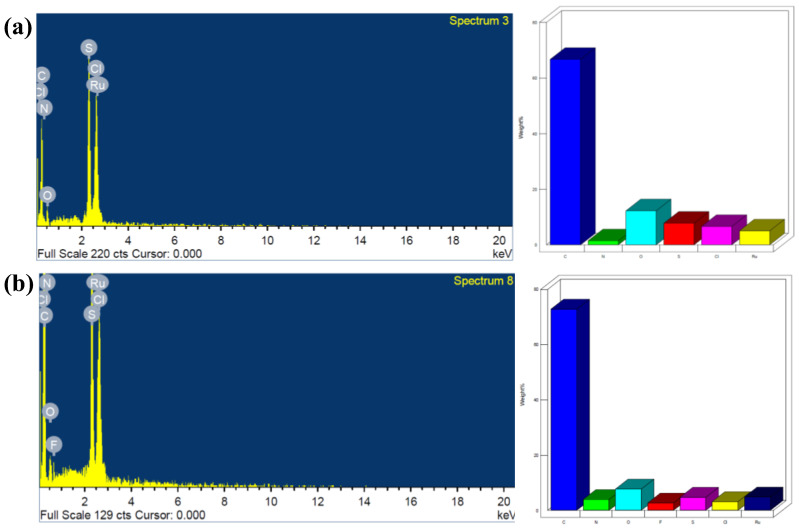
EDX images of (**a**) GO-NCD-**1** and (**b**) GO-NCD-**2**.

**Figure 6:**
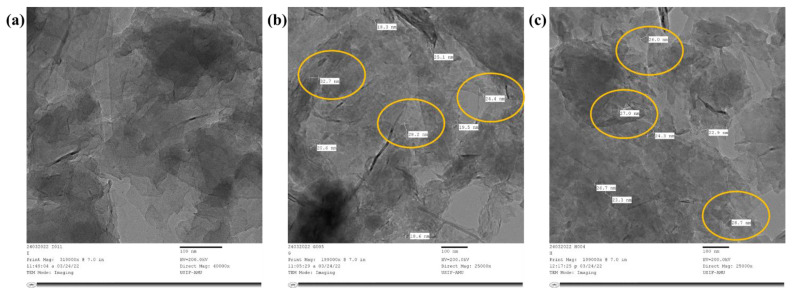
TEM images of (**a**) GO (**b**) GO-NCD-**1** and (**c**) GO-NCD-**2**.

The SEM images of GO displayed the sheet structure and the typical wrinkle structure, which could be due to the thermal fluctuations and applied stresses. SEM images of the loaded sheets showed irregular nanoparticle morphology in both the composites, therefore clearly showing the encapsulation of nanodrug candidates on the graphene oxide sheet. TEM images provided concrete evidence of the loading of the drug nanoparticles on the GO surface, with an average particle diameter of 17 ± 6.9 nm and 25 ± 6.5 nm in GO-NCD-**1** and GO-NCD-**2,** respectively. The diameter size of nanoparticles was calculated by measuring the diameters of 16 nanoparticles of uniform size and spherical shape chosen randomly through TEM images for each nanocomposite (Table S1) [31]. Furthermore, EDS results showed the presence of Ru, N, C, O, and Cl in GO-NCD-**1** and Ru, N, C, O, Cl, and F in GO-NCD-**2,** therefore providing evidence of the successful anchoring of the drug nanoparticles onto the carrier surface (Figure 4, Figure 5 and Figure 6).

### 2.2. In Vitro DNA Interaction Studies

The interaction of metal-based chemotherapeutic drug candidates with rational therapeutic target ct-DNA in aqueous solutions at neutral pH has received continued attention in the recent past, as it provides indirect evidence for their use as potential chemotherapeutic agents and aids in the development of new innovative anticancer drugs. Spectroscopic techniques such as UV-visible and fluorescence titration experiments were employed to probe the interaction between ct-DNA and GO-NCD-**1** and GO-NCD-**2**. Their binding affinity was determined by intrinsic binding constant *K*_b_ and *K*_sv_ values. Upon increase in the ct-DNA concentration, an increase in absorption intensity ‘hyperchromism’ with a slight red shift (3 and 4 nm) was observed [32]. These results indicated that nanocomposites interacted with ct-DNA via electrostatic or groove binding modes due to the presence of two labile chloride and nitrogen atoms in the heterocyclic benzothiazole ring anchored to the ruthenium center. The intrinsic binding constants, *K*_b_ of GO-NCD-**1** and GO-NCD-**2** were quantified using the Wolfe–Shimmer Equation (1)

[DNA]/(ε_a_ − ε_f_)] = [DNA]/(ε_b_ − ε_f_)] + 1/*K*_b_(ε_b_ − ε_f_) (1)

where [DNA] is the DNA concentration ε_a_, ε_f_, and ε_b_ are the apparent (A_abs_/[GO-NCD-**1** or GO-NCD-**2**], free, and bound complex extinction coefficients, respectively. 

The *K*_b_ given by the ratio of slope to intercept of the straight-line plot of [DNA]/(ε_a_ − ε_f_) vs. [DNA] was obtained by monitoring the changes in the absorbance at the corresponding λ_max_ of the intraligand band. The Gibbs free energy value of the complex was obtained from Equation (2).

ΔG = −RT ln *K*_b_ (2)

where R = 8.314 J K^−1^ mol^−1^; T = 298 K.

**Figure 7:**
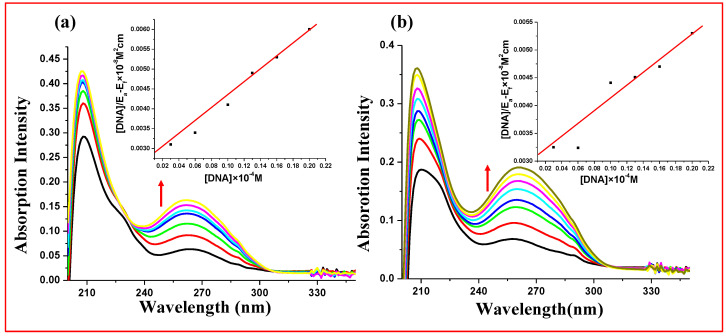
UV-visible spectra of (**a**) GO-NCD-**1** and (**b**) GO-NCD-**2**.

The results revealed a stronger binding interaction of GO-NCD-**2** with the biomolecule, ct-DNA in comparison to GO-NCD-**1**. However, these values are higher, therefore implicating a higher and better interaction of the graphene-loaded drug candidates (Table 1, Figure 7).

**Figure 8:**
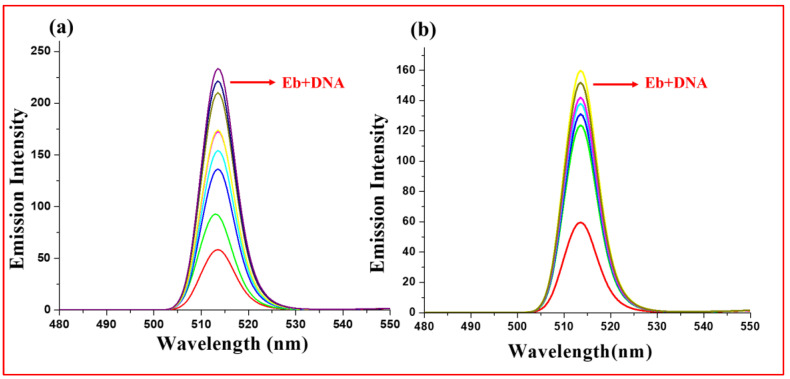
Emission spectra of (**a**) GO-NCD-**1** and (**b**) GO-NCD-**2**.

The DNA bound with ethidium bromide was titrated with increasing concentrations of GO-NCD-**1** and GO-NCD-**2** and a decrease in emission intensity was observed (Figure 8). The binding tendency of the nanocomposites to ethidium bromide-bound DNA was measured by employing the Stern Volmer Equation (3) and the *K*_sv_ values were calculated, which revealed stronger interaction of GO-NCD-**2** with the target molecule [33].

I_o_/I = 1 + *K*_sv_ [Q] (3)

where I_o_ and I represent fluorescence intensities in the absence and presence of a quencher, respectively, *K*_sv_ is the quenching constant and [Q] is the concentration of the quencher.

**Figure 9:**
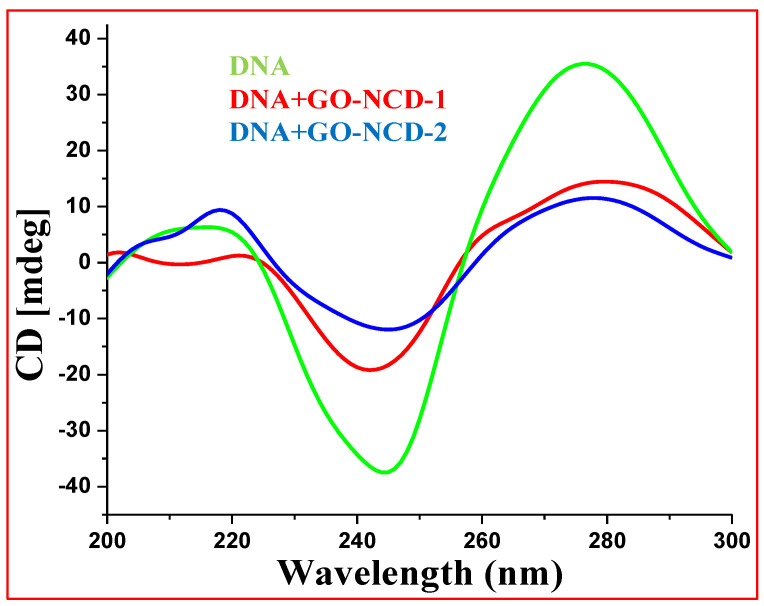
Circular dichroism spectra of GO-NCD-**1** and GO-NCD-**2** with DNA.

Circular dichroism spectroscopy was employed to diagnose the variation in the secondary structure of ct-DNA upon its interaction with nanocomposites. The CD spectrum of ct-DNA gives a maximum at 248 nm and a minimum at 275 nm which are attributed to the base stacking and right-handed helicity of the biomolecule. The spectra of both of the nanoconjugates, GO-NCD-**1** and GO-NCD-**2,** were studied with ct-DNA in a 1:1 ratio, and the change in the spectra was observed (Figure 9). The ellipticity values for both the bands changed significantly, suggesting that both of the nanoconjugates interacted with the biomolecule and modified the DNA secondary structure [34].

**Figure 10:**
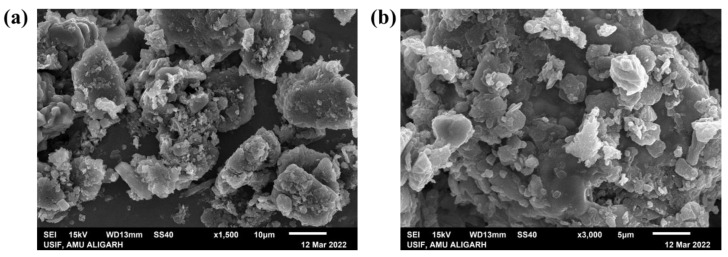
SEM images of (**a**) GO-NCD-**1** and (**b**) GO-NCD-**2** with DNA.

DNA condensates of both the GO-NCD-**1** and GO-NCD-**2** were prepared, and the changes in the morphology upon interaction with ct-DNA were studied by employing the SEM. Since transition metals are charged species, they can induce DNA condensation in vitro and change the morphology of DNA into bundles, rodlike shapes, toroids, charged globules, and segregated chains, depending upon solution conditions (Figure 10). The results suggested that interaction of the DNA molecules upon condensation with nanoconjugates resulted in the formation of globular structures, which indicated the presence of charged surfaces [35].

### 2.3. DNA Cleavage Studies by Electrophoretic Gel Assay

**Figure 11:**
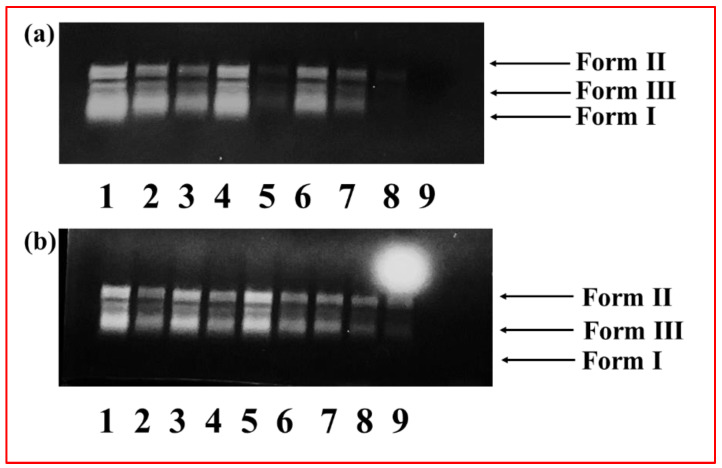
Plasmid DNA cleavage activity of (**a**) GO-NCD-**1** and (**b**) GO-NCD-**2** at different concentrations, 2.5 μM to 20 μM (lane 2 to 9) (Lane 1 = DNA control).

The change in electrophoretic mobility of pBR322 plasmid DNA on agarose gel provided further evidence for DNA-drug interactions [36,37,38]. The migration of pBR322 plasmid DNA from its supercoiled form (Form I) to its nicked (Form II) or linear form (Form III) after its interaction with drug candidates provides evidence for DNA-drug interactions [39]. To comprehend the cleaving capacity of GO-NCD-**1** and GO-NCD-**2**, in an aqueous saline Tris-HCl buffer (7.4 pH) a change in the electrophoretic mobility of pBR322 plasmid DNA was observed. The results showed impressive cleavage at 7.5 and 10 µM concentrations in GO-NCD-**1** and GO-NCD-**2** and the appearance of the linearized form (form III) of DNA in both cases was indicative of more lethal double-stranded cleavage (Figure 11).

**Figure 12:**
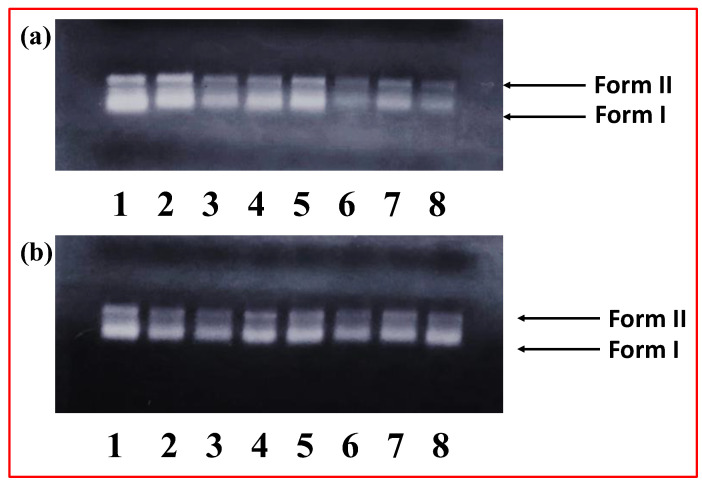
Plasmid DNA cleavage activity of (**a**) GO-NCD-**1** and (**b**) GO-NCD-**2**. Lane 1 = DNA control. Lane 2–8 (EtOH, DMSO, NaN_3_, SOD, MG, H_2_O_2_, and GSH).

To understand the mechanism of cleavage, the cleaving activity of plasmid DNA was investigated in the presence of reactive oxygen species (ROS) such as EtOH and DMSO, singlet oxygen scavengers ^1^O_2_ (NaN_3_ and SOD), and groove binding agents such as methyl green (Figure 12). From the results, it was concluded that EtOH and DMSO do not inhibit the cleavage process, suggesting non-involvement of (^.^OH) radicals, and a slight enhancement in the SC (form I) was observed in the presence of groove binding agents methyl green and NaN_3_, therefore suggesting the involvement of reactive oxygen species in DNA cleavage, which further supported the observed spectroscopic results [40,41].

### 2.4. Computational Studies

**Figure 13:**
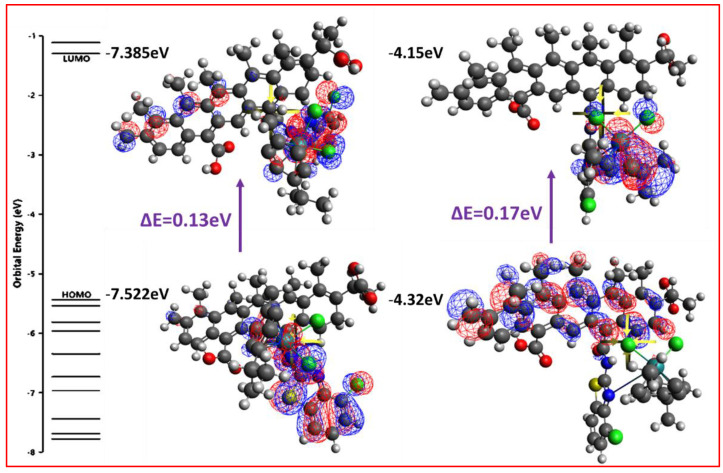
Frontier molecular orbital surfaces of GO-NCD-**1** and GO-NCD-**2**.

DFT calculations were carried out with Orca software, and the calculation of the orbital energies was done by using the BP basis set as *E*_HOMO_ and *E*_LUMO_ energies play key roles in the prediction of chemical reactivity. The computational investigations revealed that the HOMO and LUMO energies for GO-NCD-**1** and GO-NCD-**2** were equal to −7.522 eV, −4.32 eV and −7.385 eV, −4.15 eV, respectively, and the energy gap was found to be 0.13 and 0.17 eV, respectively, which indicated the stability of the nanoconjugates [42]. The calculated energy gap was found to be lower than complexes alone, therefore suggesting greater reactivity of the GO-NCD-**1** and GO-NCD-**2,** which predict the fast release of drug candidates from GO, thereby increasing their target-specific therapeutic efficacy (Figure 13).

**Figure 14:**
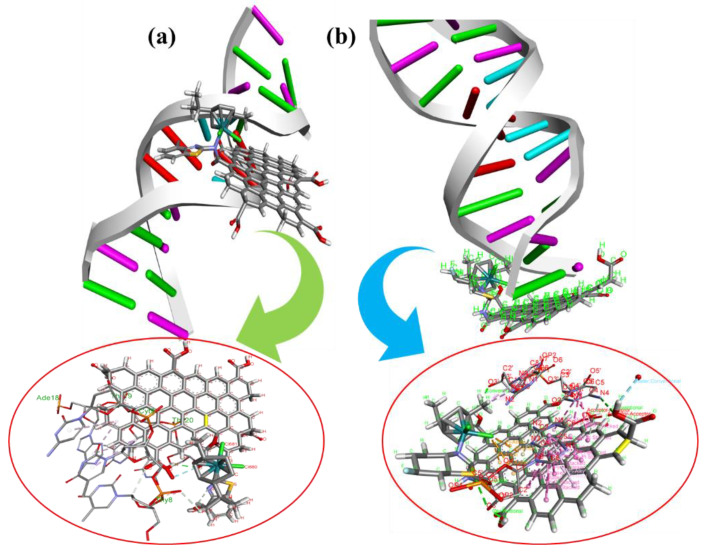
Docked pose of (**a**) GO-NCD-**1** and (**b**) GO-NCD-**2**.

Docking studies play an important part in understanding the interaction between drug candidates and the active site of a receptor [43]. Thus, molecular docking analysis was performed to elucidate the binding of GO-NCD-**1** and GO-NCD-**2** at the target site of the DNA duplex in PDB ID 1bna [44]. The binding energy scores for the GO-NCD-**1** and GO-NCD-**2** were found to be −276.25 kJ mol^−1^ and −309.88–276.25 kJ mol^−1^ which implicated a stronger binding interaction of GO-NCD-**2** than GO-NCD-**1** with the ct-DNA which in turn is greater than the complexes alone, suggesting potent binding affinity of the loaded nano drug conjugates. This indicated that the graphene oxide loading of ruthenium complexes enhanced the binding affinity of the free complexes toward the examined target, ct-DNA (Figure 14). 

### 2.5. Drug Loading and Release Profile

An important parameter to evaluate the performance of a drug carrier is its drug loading capacity. UV-visible spectroscopy was used to calculate the drug loading capacity of the GO. Both the complexes NCD **1** and NCD **2** were found to exhibit strong absorbance in the 250 nm region, and this wavelength was used to calculate the percentage of drug loaded onto the carrier (Figure S9). In the loading experiment, 100 µL to 1000 µL of 10^−3^ M NCD **1** and NCD **2** were added continuously to 1 mL of 10^−3^ M GO followed by ultrasonication, and a decrease in the absorbance intensity of the complexes was observed up to 800 µM and 600 µM, respectively, after that, no significant change in intensity was observed, suggesting a maximum loading of the drug per mL of 1 mM of GO [45]. The concentration of GO was estimated from the absorbance peak at 250 nm. The 80% and 60% encapsulation for both the complexes were calculated using the Equation (4):

(4) Encapsulation capacity=(Amount of Drug loaded on GO ÷ Total amount of drug) × 100

**Figure 15:**
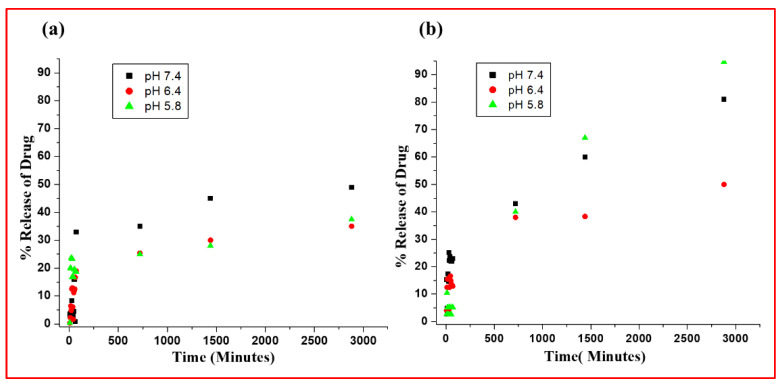
pH response of (**a**) GO-NCD-**1** and (**b**) GO-NCD-**2**.

**Figure 16:**
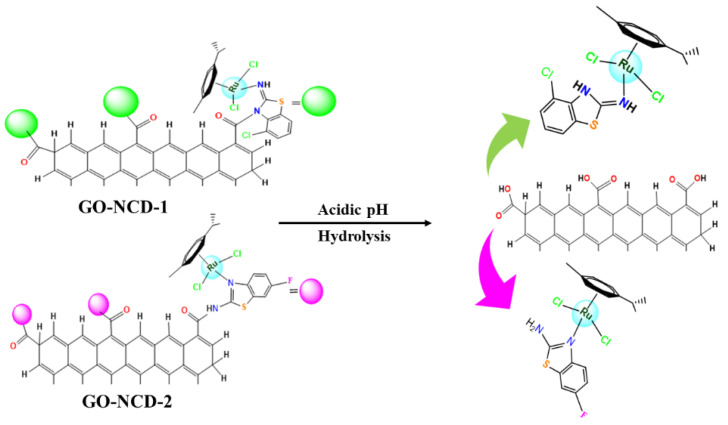
Proposed drug release mechanism.

The release behavior of the drug was studied at pH values of 7.4 (physiological pH), 6.4, and 5.8 (acidic) up to 24 h. Initially, slow release of the drug (GO-NCD-**2**) was observed, but with time, the ability to release the drug was higher at an acidic pH when compared to physiological pH (Figure 15 and Figure 16). The percentage of drug release was calculated using the Equation (5):

(5) Drug release=(Amount of released drug ÷Amount of loaded drug)×100

Initially, fast release of the drug was observed at pH 5.8 for GO-NCD-**1** up to 55 min; however, after 1 h drug was released fast at physiological pH. In the case of GO-NCD-**2,** percent drug release was almost equal at pH 7.4 and 5.8 for up to 12 h. After 12 h percent drug release was fast at an acidic pH 5.8 which could be caused by the hydrolysis under acidic conditions. This better release of drug from the nanocarrier in a slightly acidic environment is favored since cancer cells are found to have a slightly lower pH than normal cells [46,47]. It is important to note that the pH responsive behavior of the nanocarrier could have a positive impact on the controlled delivery of the drug [48].

### 2.6. Cytotoxicity

**Figure 17:**
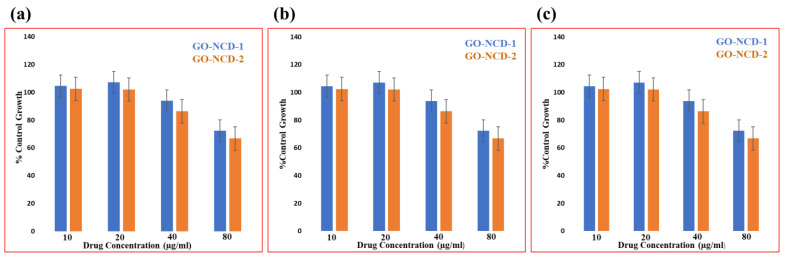
In vitro antitumor activity of GO-NCD-**1** and GO-NCD-**2** against (**a**) Human breast cancer cell line, MCF-7 (**b**) Human breast cancer cell line, MDA-MB-231 (**c**) Human cervical cancer cell line, HeLa.

Ru(II) complexes are well known for their low toxicity, diverse mechanisms of action, and non-cross resistance. The cytotoxic activity of GO-NCD-**1** and GO-NCD-**2** was assessed against human breast and cervical cancer cell lines, *Viz*. MCF7, MDA-MB-231, and HeLa, in terms of the GI_50_ by SRB assay (Figure 17, Tables S2 and S3). Earlier, the cytotoxic activity of NCD **1** and NCD **2** was carried out against the same cancer cell lines, and both the complexes showed moderate cytotoxicity, which could be due to the better hydrophobic interaction of the *p*-cymene moiety of the metal complexes with the cell membrane [23]. Moderate cytotoxicity of ruthenium complexes has been previously reported in the literature by Ivanović et al. for the [(*η*6-*p*-cymene)RuCl(picolinic acid)]H_2_O complex with an IC_50_ equal to 81.97 μM. The nanocomposites showed differences in cytotoxic profiles against the MCF-7 cell line, with better activity of GO-NCD-**2** at the 80 µg/mL concentration. In the case of the MDA-MB-231 cell line nearly identical behavior was observed at all concentrations. Nanocomposite GO-NCD-**2** was found to show good activity against the HeLa cell line at a concentration of 80 µg/mL and a more pronounced difference in the activities of GO-NCD-**1** and GO-NCD-**2** was observed against this cancer cell line. However, we conclude that the cytotoxic data suggested better and more selective activity of GO-NCD-**2** against the MCF-7 cancer cell line than GO-NCD-**1** as 60% of this drug was found loaded onto graphene oxide whereas for GO-NCD-**1**, 80% was the recorded encapsulation [49,50,51,52]. It has been reported in the literature that ruthenium arene complexes exhibit good cytotoxicity against cancerous cell lines such as MCF-7 and HeLa and low toxicity towards normal cell lines at very high doses up to 250 µM [53,54,55,56].

## 3. Materials and Method

### 3.1. Materials

All chemical reagents such as 2-amino-4-cholorobenzothiazole, 2-amino-6-fluorobenzothiazole, [Ru(ƞ6-*p*-cymene)Cl_2_]_2_, Ethidium bromide(EB), Tris-(hydroxymethyl)aminomethane(Tris-buffer), and Graphene oxide were purchased from Sigma Chemicals Co. All solvents were purchased from Merck and were used as such, without further purification.

### 3.2. Instrumentation

FT-IR spectra were recorded on a Perkin Elmer FT-IR spectrometer (PerkinElmer, Inc., Waltham, MA, USA) in the 4000–400 cm^−1^ range with KBr. PerkinElmer Lambda 25 (PerkinElmer, Inc.) was used to record electronic spectra using cuvettes of 1 cm path length. Emission spectra were obtained on a Shimadzu RF-5301 PC spectrofluorometer (Shimadzu, 1, Nishinokyo-Kuwabara-cho, Nakagyo-ku, Kyoto, Japan). Circular dichroism (CD) measurements were carried out on a Jasco J-815-CD spectropolarimeter (JASCO Corporation. 2967-5 Ishikawamachi Hachioji-shi Tokyo, Japan) using a 1 cm quartz cuvette. SEM micrographs were recorded with a JEOL JSM-6510LV scanning electron microscope (Akishima, Tokyo, Japan). Electrophoretic assays were carried out in Tris-borate-ethylenediaminetetraacetic acid buffer at 50 V cm^−1^ and visualized after incubation using a UV Transilluminator (Eppendorf, Germany). Molecular docking studies were performed using the HEX 8.0 software (Marseille, France) and visualized using the Discovery Studio molecular graphics program (Biovia Discovery Studio 2020, Dessault Systemes, Berkeley, CA, USA). The DFT studies were performed using orca. X-ray diffractograms (XRD) were obtained in the 2θ range of 5–80° with a scan rate of 8°/min on a Rigaku Minifax X-ray diffractometer (Rigaku Tokyo, Japan) with Ni-filtered Cu Ka radiation at a wavelength of 1.54060 Å.

### 3.3. Synthesis

The synthesized nanoconjugates were loaded on the graphene oxide by mixing 0.05 g of GO and 0.05 g of drug nanoconjugate (NCD 1 and NCD 2) in an aqueous dispersion (100 mL) and stirred for 12 h. The reaction mixture was sonicated for 10 min at intervals, and subsequently, centrifugation was performed [57].

### 3.4. Cytotoxicity

The cell lines MCF-7 (obtained from NCI, USA; NCCS, Pune, India), MDA-MB-231 (obtained from NCI, USA; NCCS, Pune, India) and HeLa (obtained from NCI, USA; NCCS, Pune, India) were grown in RPMI 1640 medium containing 10% fetal bovine serum and 2 mM L-glutamine. Experimental drugs were solubilized in an appropriate solvent at 100 mg/mL, diluted to 1 mg/mL using water, and stored frozen prior to use. At the time of drug addition, an aliquot of frozen concentrate (1 mg/mL) was thawed and diluted to 100 μg/mL, 200 μg/mL, 400 μg/mL, and 800 μg/mL with complete medium containing the test article. Percent Growth was expressed as the ratio of the average absorbance of the test well to the average absorbance of the control wells × 100.

## 4. Conclusions

In summary, two ruthenium-based metal complexes were loaded onto graphene oxide by ultrasonication, and the encapsulation was confirmed by employing FT-IR, UV-visible, ^1^**H** NMR, TGA, SEM, and TEM, which firmly established the successful loading of the drug candidates on the nano carrier with a particle diameter size of 17 ± 6.9 nm and 25 ± 6.5 nm. In vitro ct-DNA binding investigation of the nanocomposites GO-NCD-**1** and GO-NCD-**2** was carried out by employing UV-visible, fluorescence, and CD with results showing stronger binding interactions of the nanocomposites with the target biomolecule with high *K*_b_ and *K*_sv_ values. Both of the nanocomposites GO-NCD-**1** and GO-NCD-**2** showed effective cleavage of the pBR322 plasmid DNA at a lower concentration of 7.5 and 10 µM mediated via an oxidative pathway. DFT was employed to get an insight into the electronic structure of GO-NCD-**1** and GO-NCD-**2** and the results displayed better reactivity of the nanocomposites that suggested an enhancement in the binding affinity of the nanocomposites with the target biomolecule. Molecular docking was performed to explore the binding of the nanocomposites with the DNA, which showed stronger binding affinity of the loaded drug candidates than the free complexes, therefore complementing the spectroscopic results. Cytotoxicity studies were also performed against human breast and cervical cancer cell lines by the SRB assay, which showed moderate activity of both the nanocomposites. The drug loading capacities of GO-NCD-**1** and GO-NCD-**2** were determined and found to be 80% and 60% respectively. Fast release of drug at acidic pH can have a good impact on the controlled delivery of drug therefore, the drug release behavior of the nanocomposites was investigated at pH 7.4, 6.4, and 5.8 and better release of GO-NCD-**2** was observed at acidic pH in 48 h which showed that the release of the drug candidates from the nanocarrier is pH responsive. Therefore, this promising and conveniently prepared graphene oxide loaded nanocomposite with good pH sensitivity and better in vitro DNA interaction may find potential applications in pH-controlled drug delivery.

## Figures and Tables

**Table 1:** Calculated ***K***_b_ and ***K***_sv_ values of GO-NCD-**1** and GO-NCD-**2**.

Nanocomposite	*K*_b_ (M^−1^)	ΔG (kJ mol^−1^)	*K*_sv_ (M^−1^)
GO-NCD-1	0.57 × 10^4^ (±0.0416) M^−1^	−21.40 (±1.782) kJ mol^−1^	0.168 × 10^3^ (±0.01) M^−1^
GO-NCD-2	0.32 × 10^5^ (±0.02) M^−1^	−25.7 (±0.351) kJ mol^−1^	0.153 × 10^3^ (±0.014) M^−1^

## Data Availability

The data presented in this study are available on request from the corresponding author.

## Supplementary Materials

The following supporting information can be downloaded at: https://www.mdpi.com/article/10.3390/molecules27217592/s1, The experimental section; Figure S1: UV-visible spectra of NCD-GO-**1**; Figure S2: UV-visible spectra of NCD-GO-**2**; Figure S3: UV-visible spectra of GO; Figure S4: XRD pattern of complex **1** (NCD **1**); Figure S5: XRD pattern of complex **2** (NCD **2**); Figure S6: XRD pattern of complex GO; Figure S7: TGA of complex 1 (NCD 1); Figure S8: TGA of complex **2** (NCD **2**); Figure S9: TGA of complex 2 (NCD 2); Figure S10: ^1^H NMR spectra of (a) GO-NCD-**1** and (b) GO-NCD-**2**; Figure S11: ^1^H NMR spectra of (a) NCD 1 and (b) NCD 2.;Table S1: Average particle diameter size data of both the nano composites; Table S2: in vitro antitumor activity; Table S3: in vitro antitumor activity data.

## Abbreviations

TGA, Thermal gravity analysis; GO, Graphene oxide; EDX, Energy dispersive X-ray spectroscopy; SEM, Scanning electron microscopy.
