# Supplementary material for: Ru(II)(ƞ6-p-cymene) Conjugates Loaded onto Graphene Oxide: An Effective pH-Responsive Anticancer Drug Delivery System
Source: Molecules. 2022 Nov 5;27(21):7592. doi: 10.3390/molecules27217592 (PMC9655566; doi:10.3390/molecules27217592)
Supplement: Supplementary file 1 [file molecules-27-07592-s001.zip › molecules-1984146-supplementary.pdf]

Supplementary information

Ru(II)(η_6-*p*-cymene) Conjugates Loaded onto Graphene Oxide: An Effective pH-Responsive Anticancer Drug Delivery System

Suffora Akhter [1], Farukh Arjmand [1], Claudio Pettinari [2] and Sartaj Tabassum [1,*]

[1] Department of Chemistry, Aligarh Muslim University, Aligarh 202002, India
[2] School of Pharmacy, University of Camerino, Via S. Agostino 1, 62032 Camerino, MC, Italy
* Correspondence: tsartaj62@yahoo.com; Tel.: +91-9358255791

S1. Experimental section

S1.1. Cytotoxicity

The cell lines were grown in RPMI 1640 medium containing 10% fetal bovine serum and 2 mM L-glutamine. For present screening experiment, cells were inoculated into 96 well microtiter plates in 100 µL at plating densities as shown in the study details above, depending on the doubling time of individual cell lines. After cell inoculation, the microtiter plates were incubated at 37° C, 5 % CO2, 95 % air and 100 % relative humidity for 24 h prior to addition of experimental drugs. Experimental drugs were solubilized in appropriate solvent at 100mg/ml and diluted to 1mg/ml using water and stored frozen prior to use. At the time of drug addition, an aliquote of frozen concentrate (1mg/ml) was thawed and diluted to 100 µg/ml, 200 µg/ml, 400 µg/ml and 800 µg/ml with complete medium containing test article. Aliquots of 10 µl of these different drug dilutions were added to the appropriate microtiter wells already containing 90 µl of medium, resulting in the required final drug concentrations i.e.,10 µg/ml, 20 µg/ml, 40 µg/ml, 80 µg/ml.

After compound addition, plates were incubated at standard conditions for 48 hours and assay was terminated by the addition of cold TCA. Cells were fixed *in situ* by the gentle addition of 50 µl of cold 30 % (w/v) TCA (final concentration, 10 % TCA) and incubated for 60 minutes

at 4°C. The supernatant was discarded; the plates were washed five times with tap water and air dried. Sulforhodamine B (SRB) solution (50 µl) at 0.4 % (w/v) in 1 % acetic acid was added to each of the wells, and plates were incubated for 20 minutes at room temperature. After staining, unbound dye was recovered and the residual dye was removed by washing five times with 1 % acetic acid. The plates were air dried. Bound stain was subsequently eluted with 10 mM trizma base, and the absorbance was read on a plate reader at a wavelength of 540 nm with 690 nm reference wavelength.

Percent growth was calculated on a plate-by-plate basis for test wells relative to control wells. Percent Growth was expressed as the ratio of average absorbance of the test well to the average absorbance of the control wells * 100.

Using the six absorbance measurements [time zero (Tz), control growth (C), and test growth in the presence of drug at the four concentration levels (Ti)], the percentage growth was calculated at each of the drug concentration levels. Percentage growth inhibition was calculated as:

[Ti/C] x 100 %

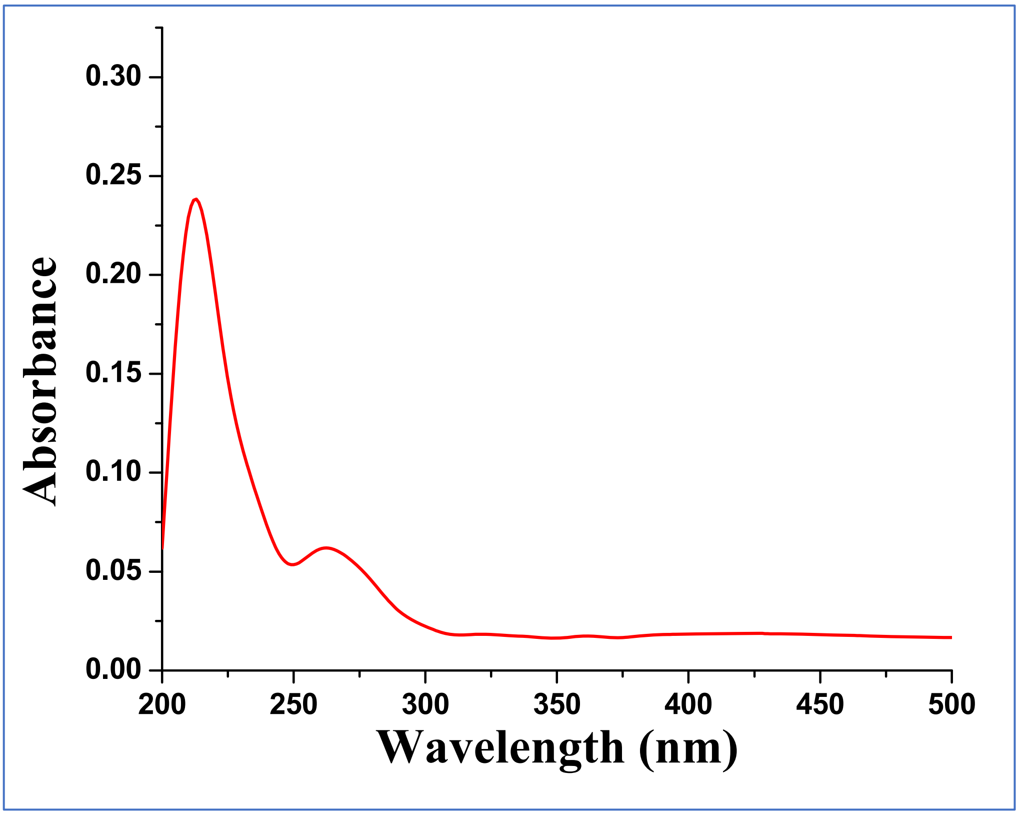

Figure S1. UV-visible spectra of NCD-GO-**1**

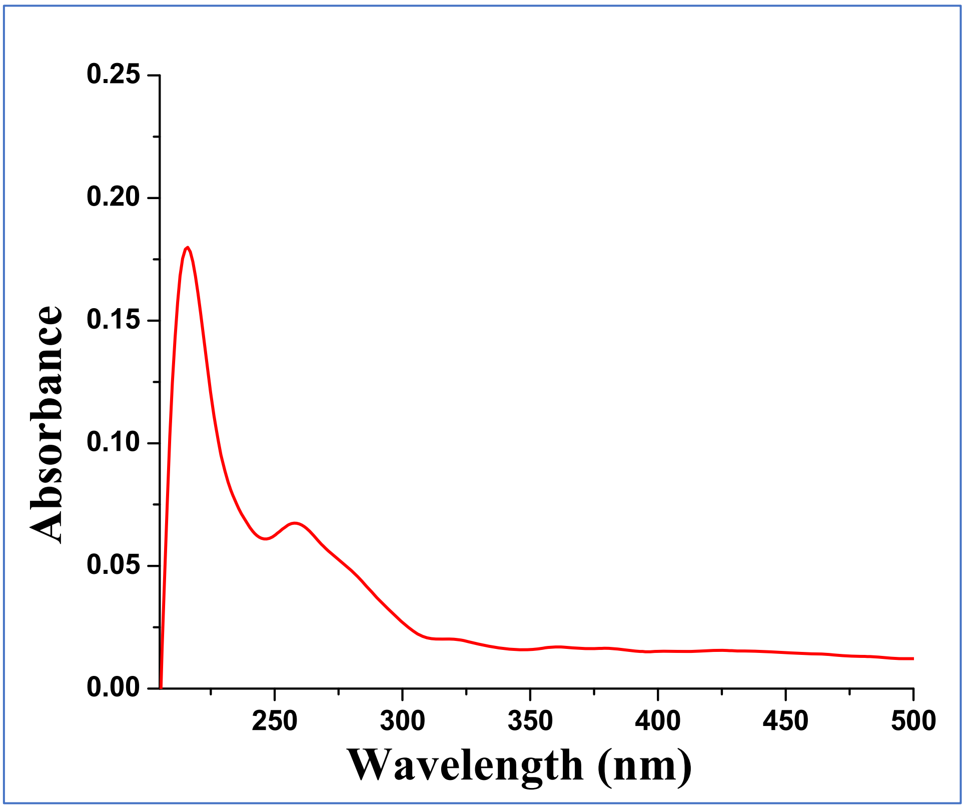

Figure S2. UV-visible spectra of NCD-GO-**2**

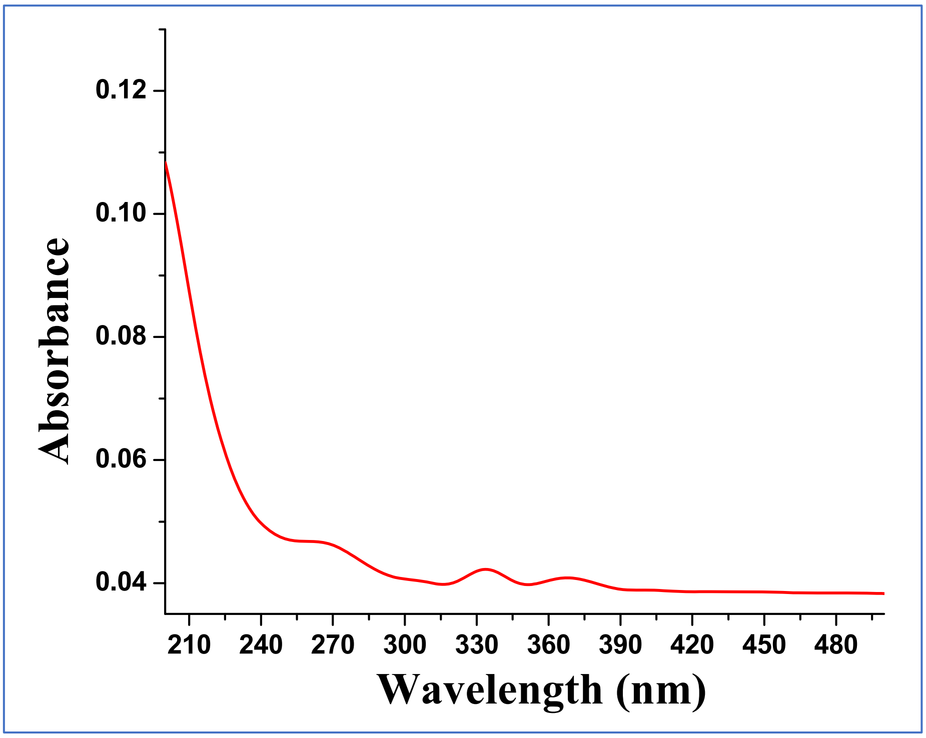

Figure S3. UV-visible spectra of GO

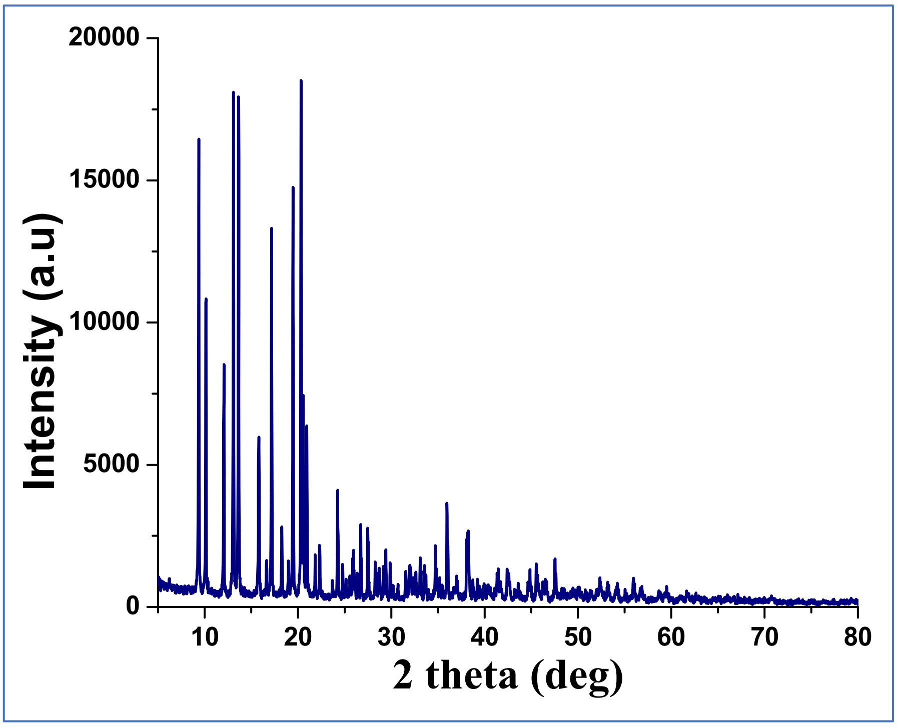

Figure S4.XRD pattern of complex **1** (NCD **1**)

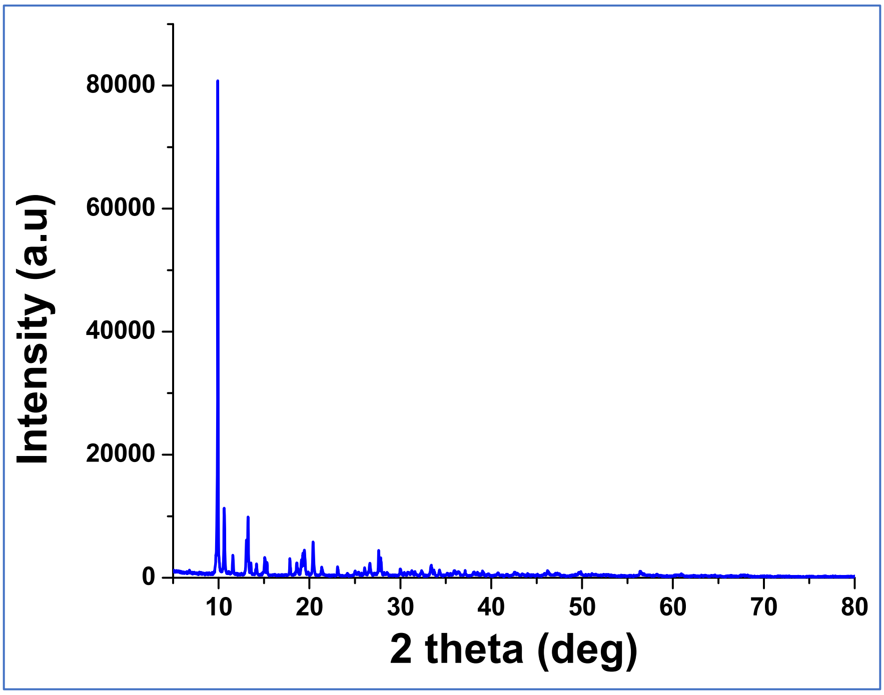

Figure S5.XRD pattern of complex **2** (NCD **2**)

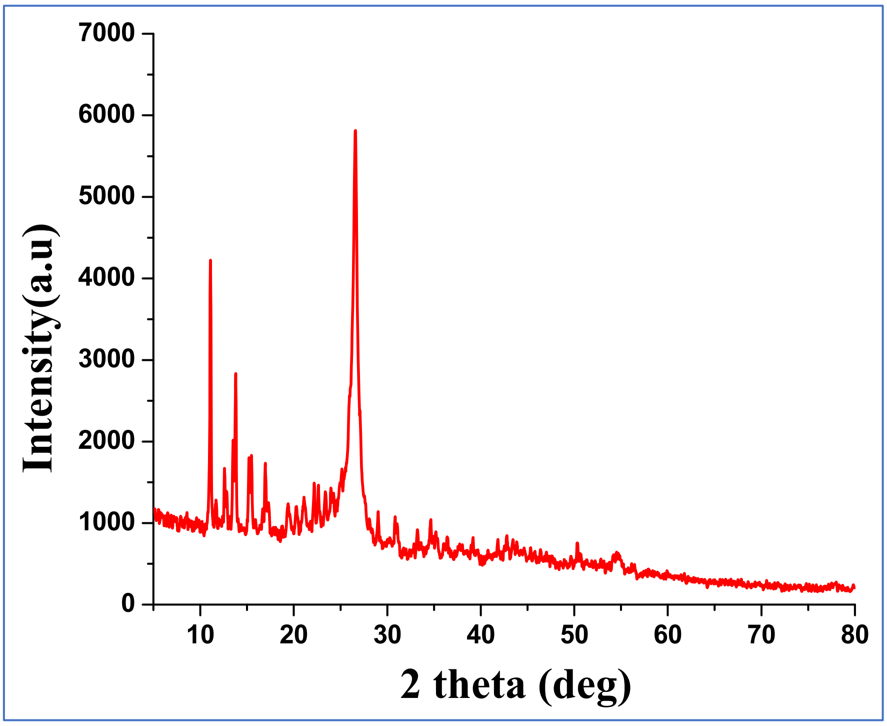

Figure S6. XRD pattern of complex GO

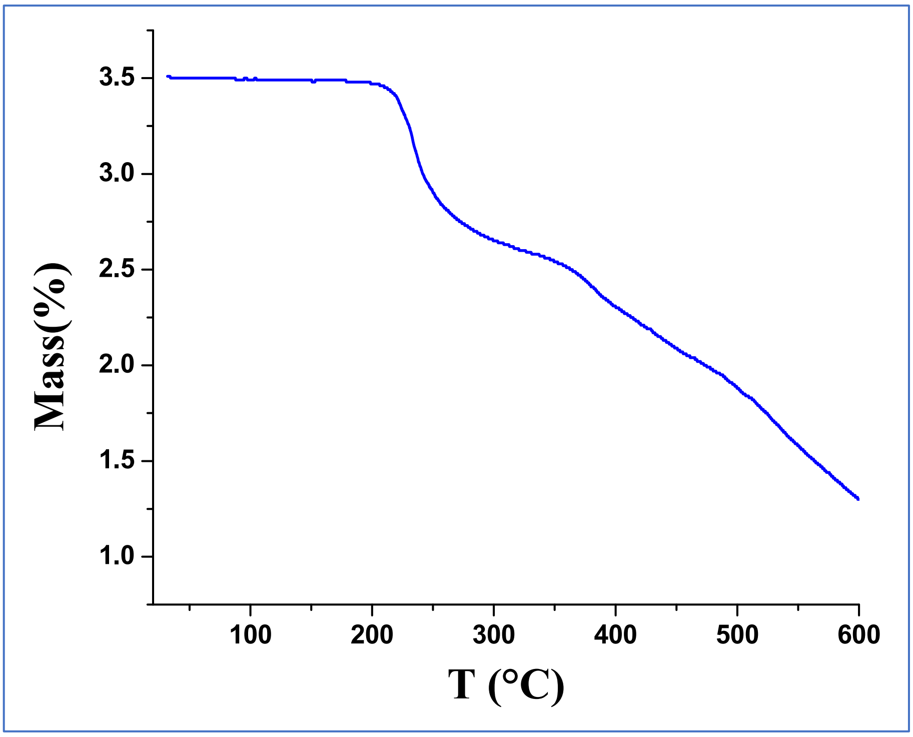

Figure S7. TGA of complex **1** (NCD **1**)

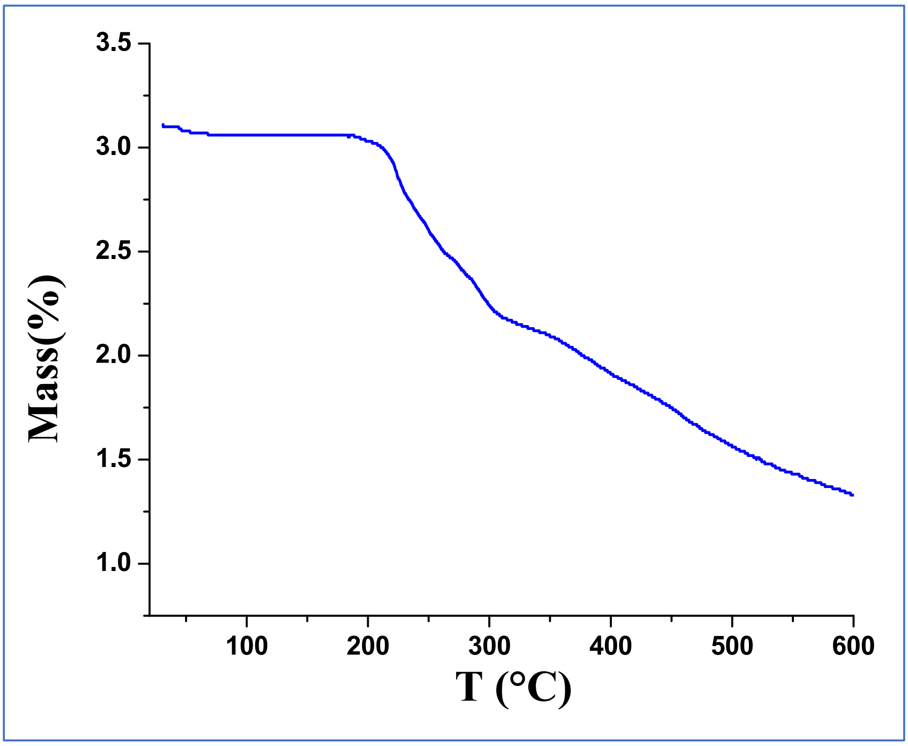

Figure S8. TGA of complex **2** (NCD **2**)

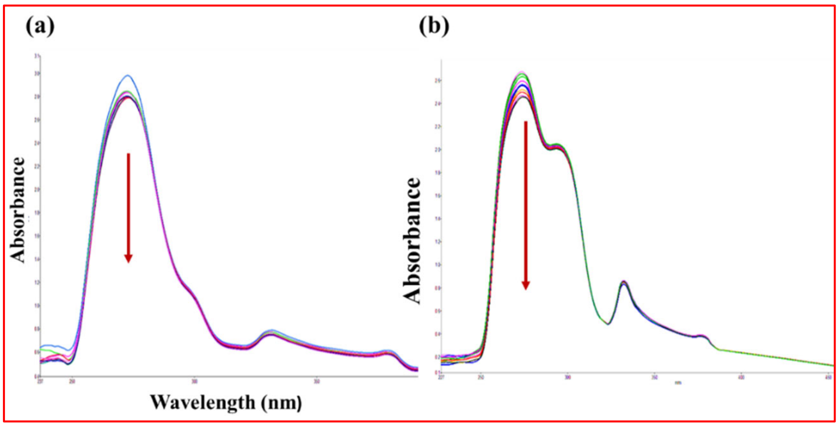

Figure S9. Drug loading graph of (a) NCD-GO-**1** and (b) NCD-GO-**2**

Table S1. Average particle diameter size data of both the nano composites.

S. No	GO-NCD-1	Go-NCD-2
1	18.3	31.6
2	25.1	24.4
3	32.7	40.6
4	28.2	26.5
5	20.6	30.8
6	24.4	29.3
7	19.5	26.0
8	18.6	27.0
9	7.96	24.3
10	16.1	22.9
11	10.6	26.7
12	13.6	23.3
13	11.3	28.7
14	17.9	14.6
15	10.0	15.3
16	13.4	16.3
Average	17.99	25.51
Standard Deviation	6.99	6.55

Table S2. *in vitro* antitumor activity.

Complex	MCF-7	MDA-MB-231	HeLa
GO-NCD-1	>80	>80	>80
GO-NCD-2	>80	>80	>80
ADR	<10	<10	<10
Cisplatin	23.59±2.50	-	-

Table S3. *in vitro* antitumor activity data.

	Human Breast Cancer Cell Line MCF-7															
	% Control Growth															
	Drug Concentrations (µg/ml)															
	Experiment 1				Experiment 2				Experiment 3				Average Values			
	10	20	40	80	10	20	40	80	10	20	40	80	10	20	40	80
GO-NCD-1	94.4	95.6	94.1	98.2	106.1	97.1	88.8	78.7	97.3	100.0	85.1	85.9	99.3	97.6	89.3	87.6
GO-NCD-2	99.9	99.6	96.8	80.5	104.5	97.4	87.6	67.3	95.3	96.4	88.2	69.5	99.9	97.8	90.9	72.5

	Human Breast Cancer Cell Line MDA-MB-231															
	% Control Growth															
	Drug Concentrations (µg/ml)															
	Experiment 1				Experiment 2				Experiment 3				Average Values			
	10	20	40	80	10	20	40	80	10	20	40	80	10	20	40	80
GO- NCD-1	97.1	97.7	95.4	108.1	79.8	88.9	92.9	82.1	100.2	107.2	93.8	82.1	92.4	98.0	94.0	90.7
GO – NCD-2	96.9	107.7	106.8	117.4	86.5	87.8	84.3	86.0	99.1	98.4	90.7	75.7	94.1	98.0	93.9	93.0

	Human Cervical Cancer Cell Line HeLa															
	% Control Growth															
	Drug Concentrations (µg/ml)															
	Experiment 1				Experiment 2				Experiment 3				Average Values			
	10	20	40	80	10	20	40	80	10	20	40	80	10	20	40	80
GO- NCD-1	98.5	102.8	93.9	73.8	106.7	108.1	97.8	75.4	108.2	110.4	89.8	67.6	104.5	107.1	93.8	72.3
GO – NCD-2	97.5	95.9	85.3	63.5	104.5	102.8	91.6	68.6	105.1	107.4	82.0	68.2	102.4	102.0	86.3	66.8

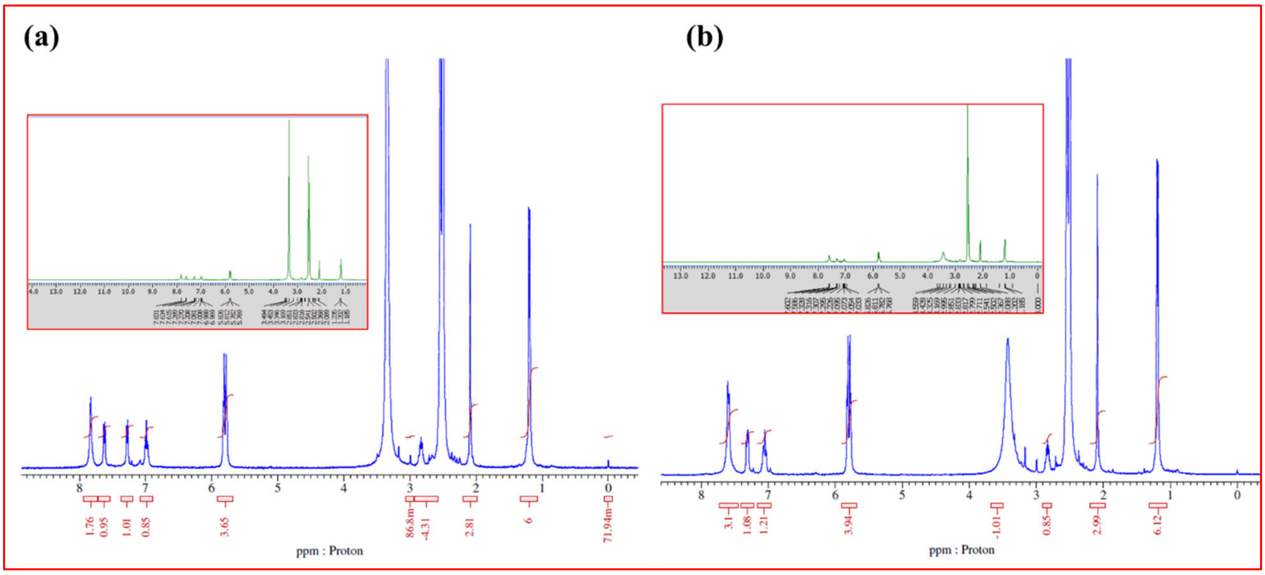

Figure S10. **^{1}H** NMR spectra of (a) GO-NCD-**1** and (b) GO-NCD-**2**

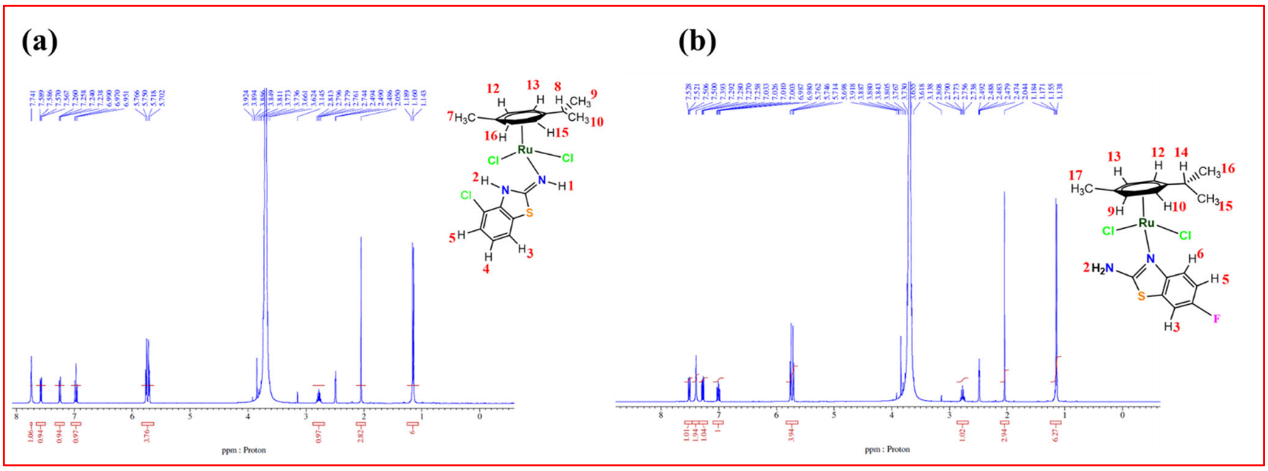

Figure S11. **^{1}H** NMR spectra of (a) NCD **1** and (b) NCD **2**.
